# Electroacupuncture Improves IBS Visceral Hypersensitivity by Inhibiting the Activation of Astrocytes in the Medial Thalamus and Anterior Cingulate Cortex

**DOI:** 10.1155/2020/2562979

**Published:** 2020-06-12

**Authors:** Min Zhao, Zhaoqin Wang, Zhijun Weng, Fang Zhang, Guona Li, Zhe Ma, Huangan Wu, Yuhu Xin, Huirong Liu, Jimeng Zhao

**Affiliations:** ^1^Key Laboratory of Acupuncture and Immunological Effects, Shanghai University of Traditional Chinese Medicine, Shanghai 200030, China; ^2^Shanghai Research Institute of Acupuncture and Meridian, Shanghai 200030, China; ^3^Fudan University Shanghai Cancer Center, Shanghai 200032, China

## Abstract

**Objective:**

To explore whether the effect of electroacupuncture (EA) on visceral hypersensitivity (VH) in rats with irritable bowel syndrome (IBS) is related to the changes of astrocyte activation in the medial thalamus (MT) and anterior cingulate cortex (ACC).

**Method:**

Male Sprague-Dawley rats were randomly divided into the normal control (NC) group, model control (MC) group, electroacupuncture (EA) group, and fluorocitrate (FCA) group. A model of visceral hypersensitivity was established by neonatal colorectal irritation. In the EA group, needles were inserted into the skin at the Tianshu (ST25) and Shangjuxu (ST37) acupoints, once a day for 7 days. The FCA group received intrathecal injection of FCA on the 1st, 4th, and 7th days. Visceral hypersensitivity was evaluated by the abdominal withdrawal reflex (AWR), and glial fibrillary acidic protein (GFAP) mRNA and protein levels in the MT and ACC were detected by real-time PCR, immunohistochemistry, and western blots.

**Results:**

The AWR score in the MC group was significantly higher than in the NC group, and EA and FCA reduced the AWR score of VH rats. GFAP mRNA and protein levels in the MT and ACC of rats in the MC group were significantly increased compared with the NC group. After either electroacupuncture or fluorocitrate, GFAP mRNA and protein levels in the MT and ACC were both clearly reduced.

**Conclusion:**

Electroacupuncture alleviates IBS visceral hypersensitivity by inhibiting the activation of astrocytes in the MT and ACC.

## 1. Introduction

Irritable bowel syndrome (IBS) is a common chronic functional gastrointestinal disease characterized by abdominal pain, abdominal distension, and other changes in defecation habits without structural alteration. The global prevalence of IBS is 10%, which seriously affects the quality of life [1, 2]. Visceral hypersensitivity is the most important pathophysiological feature about IBS [3, 4], but the mechanism is still unclear.

**Figure 1:**
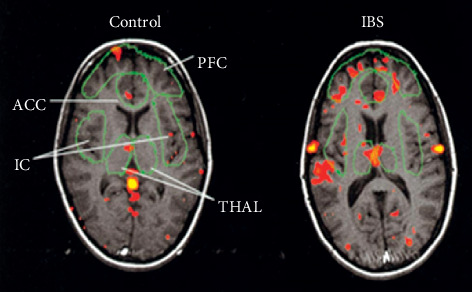
Functional magnetic resonance imaging (fMRI) shows that the activation degree of the thalamus (THAL) and ACC in patients is higher than that in normal subjects (cited from Mertz (H) Gastroenterology.2000 [6]).

Visceral nociceptive stimulation is transmitted from the primary sensory nerve to the sensory ganglion and then up to elements of the high central nervous system, such as the medial thalamus (MT) and anterior cingulate cortex (ACC), for transmission and integration [5]. In recent years, increasing research has found that the activation of the thalamus and ACC is related to visceral hypersensitivity of IBS (Figure 1) [6, 7]. IBS could affect the activation of MT and ACC brain regions and induce the corresponding synaptic signal transmission [8]. Astrocytes was found to be involved in pain signal transduction of IBS in brain regions, visceral hypersensitivity activates astrocytes in the thalamus and ACC, and the activation of astrocytes is positively correlated with the behavior results of rats with visceral pain [9, 10]. Therefore, it is believed that MT and ACC mediated by astrocytes are important brain regions involved in IBS visceral hypersensitivity.

Regulation of the intestinal flora, gastrointestinal motility, and antidepressant treatments are the common treatments for IBS, but the effect is not sufficient and there are some side effects [11]. Acupuncture is effective in treating IBS with few side effects. According to a new system review [12], electroacupuncture significantly improved the pain threshold induced by rectal expansion and improved the related clinical symptoms of IBS. ST25 and ST37 are a common acupoint combination to treat intestinal diseases [13–15]. Modern research shows that electroacupuncture at ST25 and ST37 improves visceral hypersensitivity of IBS through the brain-gut axis [16, 17], but the mechanism of electroacupuncture on visceral hypersensitivity of IBS is not very clear at present.

In this study, ST25 and ST37 were used to explore the effect of electroacupuncture on IBS visceral hypersensitivity. We speculate that the effect of electroacupuncture on IBS visceral hypersensitivity may be related to the changes of astrocyte activation in the MT and ACC and looked forward to reveal the mechanism of electroacupuncture on visceral hypersensitivity of IBS.

## 2. Materials and Methods

### 2.1. Animals

Newborn, male, specific pathogen-free- (SPF-) grade Sprague-Dawley (SD) rats (5 days old) were provided by the Experimental Animal Center of Shanghai University of Traditional Chinese Medicine (Animal License Number: SCXK (Shanghai) 2013-0016), and the neonatal rats were given free access to food and water. The housing environment was characterized by a 12 h light-dark cycle, a temperature of 20 ± 2°C, and 50–70% humidity. After adaptive feeding for 3 days, the experiments began. During the experimental process, animal treatment conformed to the guidelines of the International Association for the Study of Pain (IASP).

### 2.2. Visceral Hypersensitivity Model Establishment

Using the complete randomization method, the 8-day-old neonatal rats were divided into the normal control group and the model group. Neonatal rats in the normal control group received only genital scratching without other stimuli. For neonatal rats in the model group, we used the internationally recognized colorectal irritation method to prepare the IBS model [18]. Colorectal irritation was performed in awake animals. During the operation, liquid paraffin was applied to the surface of each balloon. The balloon was gently inserted anally along the physiological curvature of the rectum to the descending colon. At this point, the balloon was inflated at a pressure of 60 mmHg for 1 min. The injector was slowly withdrawn, and the balloon was removed. After 30 minutes, the same stimulation was repeated. Balloon stimulation was performed once every day at a fixed time for 14 days. Rats were continuously fed until the sixth week, when experimental grouping and processing were performed.

### 2.3. Groups and Treatments

After the model was successfully established, rats in the model group were grouped using the complete randomization method into the model group, electroacupuncture group, and fluorocitrate group: (1) The normal control (NC) group rats were fed normally without colorectal irritation. (2) The model control (MC) group rats were not treated after the model had been established successfully. (3) The electroacupuncture (EA) group rats received electroacupuncture at a needling depth of 5 mm at the ST25 (bilateral) and ST37 (bilateral) acupoints. The parameters of Han's acupoint nerve stimulator (HANS) were as follows: sparse-dense wave with a frequency of 2/100 Hz, a current of 2 mA, 20 min/stimulation, and one stimulation per day. The treatment was provided for seven consecutive days. The Tianshu acupoint (ST25) is at the same level as the navel and 5 mm lateral to the anterior median line; the Shangjuxu acupoint (ST37) is 5 mm lateral to the anterior tubercle of the tibia and 15 mm below the knee joint [15]. (4) The fluorocitrate (FCA) group rats received an intrathecal injection of the astrocyte inhibitor FCA (1 *μ*M, 10 *μ*l) (Sigma, USA) on the 1st, 4th, and 7th days. AWR scores were assessed within 60 min of the last treatment to evaluate the visceral hypersensitivity. The rats were sacrificed by cervical dislocation under intraperitoneal anesthesia, and the MT and ACC were quickly isolated and stored.

### 2.4. Visceral Sensitivity Assessment

AWR scores were assessed within 60 min before and after treatment. The scoring criteria are shown in Table 1 [18]. The specific steps were as follows [19]: rats were fasted 8–12 h before AWR assessment, and the abdomen was gently touched to promote stool discharge from the colorectum before colorectal distension (CRD) stimulation. When the rat was awake, the balloon was inserted through the anus along the rectum to reach the descending colon of the rat. CRD stimulation was applied at four different levels of pressure: 20, 40, 60, and 80 mmHg. Three AWR scores were measured for each rat, with each measurement lasting approximately 20 seconds, with 4 min intervals between measurements. The mean was calculated as the final score.

### 2.5. Immunohistochemistry

GFAP was detected in the MT and ACC using immunohistochemistry. The sections were washed 3 times with phosphate-buffered saline (PBS) for 5 min and exposed to 3% H_2_O_2_ for 15 min at room temperature to inhibit endogenous peroxidases. After washing with 0.01 M PBS for 3 × 5 min, the specimens were immersed in dilute anti-GFAP antibody (Abcam, UK) and incubated at 4°C overnight. The specimens were warmed for 30 min, washed with 0.01 M PBS for 3 × 5 min, and incubated with the secondary antibody (Gene tech (Shanghai) Co., Ltd., China) at room temperature for 30 min. After washing with 0.01 M PBS for 3 × 5 min, the 3,3-diaminobenzidine (DAB) chromogen solution was added. Then, the sections were sealed with neutral gum for further observation under a light microscope. A semiquantitative analysis of the staining was performed using the Image-Pro Plus 6.0 image analysis system.

### 2.6. Western Blot Analysis

Protein tissue was extracted in RIPA lysis buffer containing complete protease inhibitor cocktail (Beyotime Institute of Biotechnology, China). The protein concentration was determined using the BCA method with a BCA protein concentration determination reagent kit (Beyotime Institute of Biotechnology, China). Then, the proteins were separated by 12% sodium dodecyl sulfate polyacrylamide gel electrophoresis and transferred to polyvinylidene fluoride membranes. After the transfer was complete, the membrane was removed, placed in an incubation box, and blocked in an appropriate amount of 5% BSA on a shaker at room temperature for 1 h. The trimmed membrane was placed in an incubator and incubated with the primary antibody against GFAP (1 : 1000 in 5% BSA) (Abcam, UK), and the bound antibody was detected with horseradish peroxidase-conjugated secondary antibody for 1 h (1 : 1000 in 5% BSA) (Beyotime Institute of Biotechnology, China). The membrane was automatically exposed to a gel documentation system. Image Lab software was used to collect images.

### 2.7. Real-Time PCR

Total RNA was extracted using TRIzol reagent (Invitrogen, USA), and the corresponding cDNA was synthesized using a superscript reverse transcriptase kit (Invitrogen, USA); the relative gene expression level was normalized using the 2-ΔΔCT method. Primers with the following sequences were used for real-time PCR: GAPDH: 5′-GGCAAGTTCAACGGCACAGT-3' (sense), 5′-ATGACATACTCAGCACCGGC-3' (antisense); GFAP: 5′-TCAATGCCGGCTTCAAAGAG-3' (sense), 5′-TTCCAGGAAGCGGACCTTCT-3' (antisense). The amplification conditions were 95°C for 2 min, 40 cycles of 94°C for 10 seconds, 60°C for 10 seconds, and 72°C for 40 seconds.

### 2.8. Statistical Analysis

All experimental data were analyzed using SPSS 24.0 and presented as mean ± SD using GraphPad Prism 8.0. If the data in each group are normal and the variance is homogeneous, they were analyzed by one-way analysis of variance (ANOVA), followed by LSD to compare between groups; if the data were normal and the variance was not homogeneous, the Games–Howell method was used; if the data did not conform to the normal distribution, the nonparametric test was used, followed by the comparison between 2 groups. *P* values < 0.05 were considered statistically significant.

## 3. Results

### 3.1. Electroacupuncture Improves Visceral Hypersensitivity in Rats with IBS

**Figure 2:**
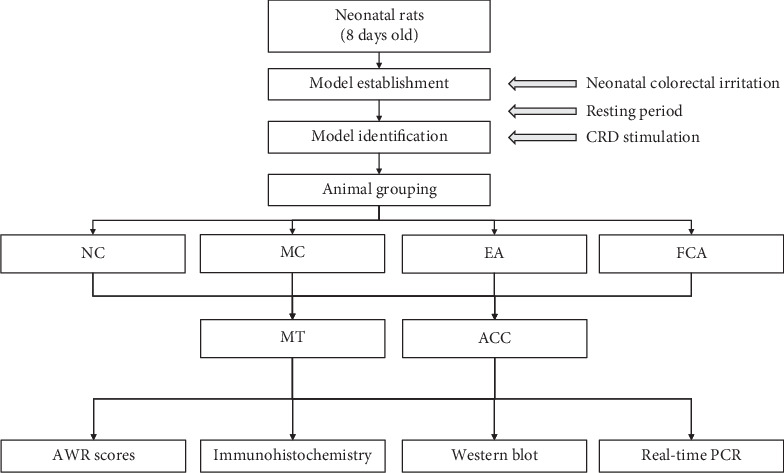
Experimental design flow chart. An IBS visceral hypersensitivity model was induced by neonatal colorectal irritation. The AWR score was used to evaluate the analgesic effect of electroacupuncture and FCA on visceral pain. Molecular biological methods were used to detect the expression of GFAP in the MT and ACC. The effect of electroacupuncture on astrocyte activation in the MT and ACC of rats with IBS visceral hypersensitivity was explored. NC: normal control group; MC: model control group; EA: electroacupuncture; FCA: fluorocitrate.

**Figure 3:**
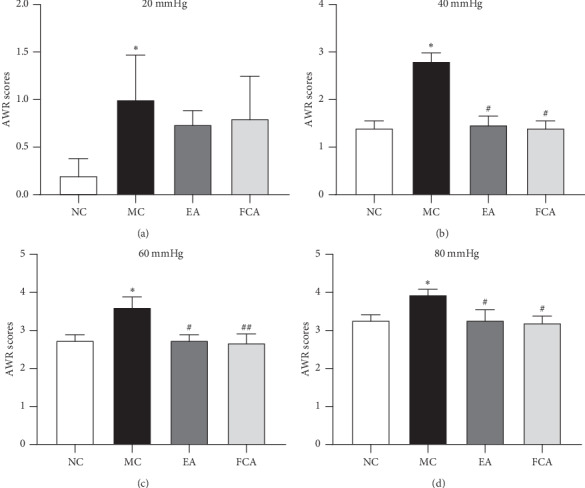
Electroacupuncture decreased AWR scores in rats with IBS with visceral hypersensitivity. Chronic visceral hypersensitivity induced by neonatal colorectal irritation continued into adulthood. Under the condition of 2/100 Hz and 1 mA current, the AWR score was significantly inhibited by electroacupuncture at the ST25 and ST37 acupoints and the analgesic effect of electroacupuncture was close to that of the intrathecal injection of FCA (1 *μ*M, 10 *μ*l). (a) AWR scores under 20 mmHg; (b) AWR scores under 40 mmHg; (c) AWR scores under 60 mmHg; and (d) AWR scores under 80 mmHg. *n* = 5, ^*∗*^*P* < 0.05 vs. NC; ^#^*P* < 0.05 and ^##^*P* < 0.01 vs. MC.

We measured the AWR score to assess visceral pain in rats with IBS and the effect of electroacupuncture on visceral hypersensitivity. As shown in Figure 2, in this study, 8-day-old neonatal rats were subjected to colorectal irritation to develop a visceral hypersensitivity model. Under a pressure stimulation of 20 mmHg, 40 mmHg, 60 mmHg, and 80 mmHg, the AWR score of the MC group was significantly higher than the NC group (*P* all ＜ 0.05) (Figures 3(a)–3(d)) and the AWR scores were increased with increasing stimulation in the MC group. After electroacupuncture and FCA, the AWR scores of rats with visceral hypersensitivity were obviously decreased. Under stimulation with 40 mm Hg, both electroacupuncture and FCA therapy significantly reduced the AWR score of rats with visceral hypersensitivity (*P* all ＜ 0.05) (Figure 3(b)). Under stimulation with 60 mmHg, the AWR scores of the EA and FCA groups were significantly lower than of the MC group (*P* < 0.05, *P* < 0.01) (Figure 3(c)). Under 80 mmHg pressure, the AWR scores of the EA and FCA groups were significantly lower than those of the MC group (*P* all ＜ 0.05) (Figure 3(d)). During the evaluation process, there was no difference in AWR scores under different levels of pressure stimulation between the EA and FCA groups (Figure 3).

### 3.2. Electroacupuncture Inhibits GFAP mRNA and Protein Levels in the MT of Rats with Visceral Hypersensitivity

**Figure 4:**
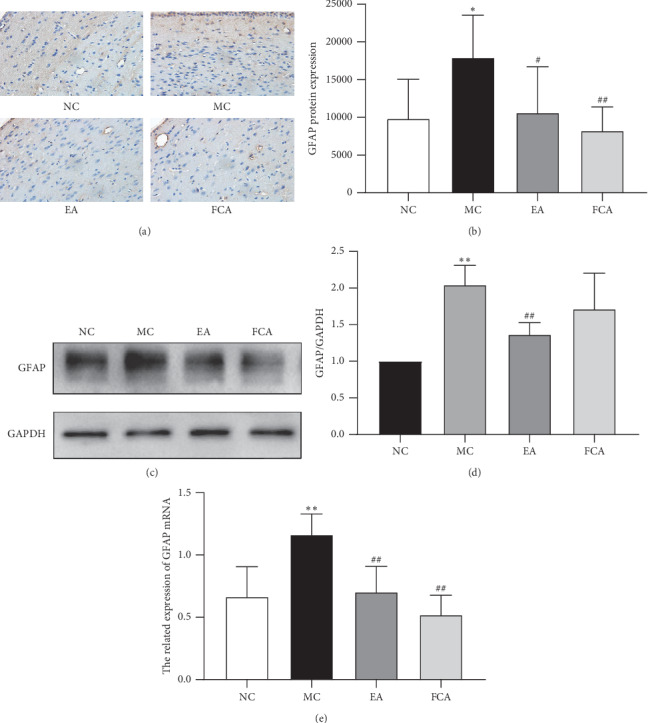
Electroacupuncture regulates GFAP gene and protein expression in the medial thalamus. The levels of GFAP mRNA and protein in the MT of rats with visceral hypersensitivity were significantly upregulated by colorectal irritation. EA and FCA clearly downregulated GFAP mRNA and protein levels in the MT of rats with visceral hypersensitivity. (a) Immunohistochemical staining of GFAP expression in MT. Scale bar: 50 *μ*m. (b) The average integrated optical density of the GFAP-positive target. (c) Representative western blot bands of GFAP in the MT. (d) Relative expression of GFAP in the MT. (e) Relative expression of GFAP mRNA in the MT. *n* = 5; ^*∗*^*P* < 0.05 and ^*∗∗*^*P* < 0.01 vs. NC; ^#^*P* < 0.05 and ^##^*P* < 0.01 vs. MC.

GFAP in the MT plays an important role in electroacupuncture for visceral hypersensitivity. As shown in Figure 4(a) and 4(b), compared with the NC group, the staining of MC group was deeper, the protrusion was thickerand longer, and the expression of GFAP was increased (*P* < 0.05). Furthermore, compared with the MC group, the staining in the EA and FCA groups was weaker, the protrusion was shorter, and the GFAP expression level was reduced (*P* < 0.05, *P* < 0.01). Western blotting showed that GFAP expression was significantly upregulated in the MT of visceral hypersensitive rats (*P* < 0.01). Electroacupuncture significantly reduced the expression of the GFAP in the MT of visceral hypersensitive rats (*P* < 0.01) (Figures 4(c) and 4(d)). As shown in Figure 4(e), the level of GFAP mRNA in the MC group was significantly increased compared to the NC group (*P* < 0.01). Both EA and FCA significantly inhibited the GFAP mRNA level in the MT of visceral hypersensitivity rats (*P* all ＜ 0.01). There was no difference in GFAP gene and protein levels between the EA and FCA.

### 3.3. Electroacupuncture Inhibits GFAP Gene and Protein Expression in the ACC of Rats with Visceral Hypersensitivity

**Figure 5:**
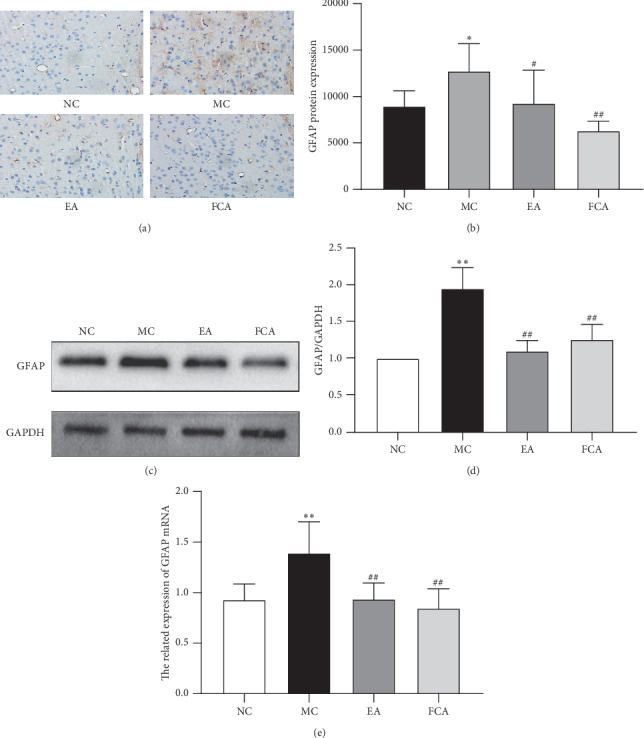
Electroacupuncture regulates the expression of the GFAP gene and protein in the anterior cingulate cortex. GFAP mRNA and protein levels were significantly increased in the ACC of rats with visceral hypersensitivity. EA and FCA therapy significantly downregulated GFAP mRNA and protein levels in the ACC of rats with visceral hypersensitivity. (a) Immunohistochemical staining showing GFAP expression in the ACC. Scale bar: 50 *μ*m. (b) The average integrated optical density of the GFAP-positive target. (c) Representative western blot bands of the GFAP in the ACC. (d) Relative expression of GFAP in the ACC. (e) Relative expression of GFAP mRNA in the ACC. *n* = 5; ^*∗*^*P* < 0.05 and ^*∗∗*^*P* < 0.01 vs. NC; ^#^*P* < 0.05 and ^##^*P* < 0.01 vs. MC.

Our results show that visceral hypersensitivity may affect GFAP levels in the ACC, which is involved in the treatment using electroacupuncture for visceral hypersensitivity. The immunohistochemical results showed that, compared with the NC group, the staining of the MC group was deeper, the protrusion was thicker and longer, and the expression of the GFAP in the ACC was increased (*P* < 0.05). Furthermore, compared with the MC group, the staining in the EA and FCA groups was more shallow, the protrusion was shorter, and the expression level of the GFAP in the ACC was lower (*P* < 0.05, *P* < 0.01) (Figure 5(a) and Figure 5(b)). Western blot analysis showed that the expression of GFAP in the ACC was significantly upregulated in the MC group (*P* < 0.01). EA and FCA therapy significantly downregulated the expression of GFAP in the ACC of rats with visceral hypersensitivity (*P* all <0.01) (Figures 5(c) and 5(d)). As shown in Figure 5(e), the GFAP mRNA level in the ACC of rats in the MC group was significantly higher than in the NC group (*P* < 0.01). The GFAP mRNA levels in the ACC of rats in the EA and FCA groups were significantly lower than those of the MC group (*P* all < 0.01). There was no difference in GFAP gene and protein levels between the EA and FCA groups.

## 4. Discussion

In this study, we found that the visceral pain threshold of rats with IBS visceral hypersensitivity was reduced, which was related to increased expression of GFAP in MT and ACC. Electroacupuncture could inhibit the expression of GFAP in MT and ACC, raise the visceral pain threshold, and relieve the visceral hypersensitivity of IBS.

The study showed that the AWR scores of visceral hypersensitivity rats were significantly increased and intestinal sensitivity was increased. Electroacupuncture is widely used to relieve pain and gastrointestinal diseases, and the effect is still adequate [20]. Many animal studies have found that electroacupuncture reduces the AWR score of IBS rats and regulates visceral hyperalgesia [21–23]. A systematic review and meta-analysis also found that electroacupuncture significantly augmented the pain threshold and relieved abdominal pain in patients with IBS [12, 24]. Our research was consistent with most of the previous studies, confirming that electroacupuncture can downregulate the AWR score and improve the colorectal pain threshold and visceral sensitivity in IBS rats. ST25 and ST37 together is a common acupoint combination used to treat intestinal diseases. ST25 is dominated by the skin branch of the T13-L2 spinal nerve, which transmits signals from colon nociceptive stimulation, and ST37 is dominated by the skin branch of the L6-S2 spinal nerve, which transmits signals from pelvic rectal stimulation [25, 26]. Modern research suggests that somatic and visceral sensory nerves interact at different levels in the peripheral and central regions [27]. IBS visceral hypersensitivity involves complex brain neural networks. Both the MT and ACC receive gastrointestinal signals from the spinal cord or vagus nerve and then integrate visceral sensation, emotion, and cognition. This study confirmed that electroacupuncture at ST25 and ST37 regulates the activation of the MT and ACC brain regions and plays an important role in IBS visceral hypersensitivity.

In the present study, electroacupuncture alleviated visceral hypersensitivity by regulating the expression of GFAP, an astrocyte marker, in the medial thalamic nucleus. The MT is an important relay station that receives visceral pain information from the spinal cord and then transmits this information to the cortex through the myelothalamic tract for integration and regulation. Clinical studies have found that activation of the thalamus is increased in patients with IBS depending on the level of rectal expansion, and electroacupuncture was shown to regulate MT activation and participate in the ascending pain transmission pathway of IBS through fMRI technology [6, 28, 29]. Astrocytes are widely distributed in brain regions, communicating with neurons and participating in the transmission of synaptic signals. Astrocytes in the thalamic intralaminar nuclei were found to be significantly activated in IBS rats, and astrocyte activation was positively correlated with visceral pain behavior [9], suggesting that thalamic astrocytes are involved in the regulation of visceral hyperalgesia signals. At present, acupuncture has been found to change the ultrastructure and functions of astrocytes in only a few brain areas, such as the hippocampus [30], but no studies have found that electroacupuncture regulates astrocytes in the MT. Therefore, this study used molecular biology techniques to find that GFAP gene and protein levels in the MT of IBS rats are increased. The decreased expression of GFAP suggests that electroacupuncture inhibits astrocyte activity in the MT of IBS rats, similar to the effects of intrathecal injection of FCA. Combined with these results, IBS visceral hypersensitivity may be related to astrocyte activation in the MT, and electroacupuncture improves visceral hypersensitivity by inhibiting the activation of astrocytes in the MT.

Next, electroacupuncture reduces visceral hypersensitivity by regulating the expression of GFAP in ACC. The ACC is a major part of the limbic system and a central area for pain transmission and emotion. Clinical studies have confirmed that the visceral hypersensitivity response in IBS patients activates the ACC brain region and is increased by enhancement of the degree of colorectal dilatation; furthermore, electroacupuncture has been confirmed to reduce voxel values in the ACC brain region and improve the pain threshold in IBS patients [31–33]. One animal research has also found that CRD stimulation induces ACC neuronal response in visceral hypersensitive rats [34], providing direct electrophysiological evidence for ACC sensitization in visceral hypersensitive rats. Our experimental results also showed that the ACC is involved in IBS visceral hypersensitivity. Astrocytes have been reported to be involved in the activation of ACC brain regions in IBS. Visceral hypersensitivity was shown to activate reactive astrocytes in the ACC, upregulate the expression of the immunoreactive markers GFAP and S100 B, and affect cognitive and decision-making functions; photogenetic techniques were applied to affect the release of neurotransmitters in ACC astrocytes to regulate visceral pain dysfunction, according to the latest study [10]. Previous studies have found that electroacupuncture alleviated pain memory by inhibiting the expression of cyclic-AMP response binding protein (CREB) in ACC astrocytes [35]. In this study, we found that GFAP gene and protein levels were significantly increased in the ACC of rats with IBS visceral hypersensitivity and electroacupuncture significantly inhibited GFAP gene and protein expression, similar to FCA therapy, which suggests that electroacupuncture significantly improved IBS visceral hypersensitivity by inhibiting astrocyte activation in ACC.

The MT can project into the ACC, which forms a circuit for pain processing [36, 37]. Studies have found that IBS visceral hypersensitivity synchronously affects MT and ACC activation, enhances MT-ACC synaptic field potential, activates the MT-ACC pathway, and upregulates the expression of gap junction protein connexion43 (Cx43) and excitatory amino acid transporter 2 (EAAT2) in astrocytes [7, 8, 38]. We have shown that the alleviation of the visceral hypersensitivity of IBS using electroacupuncture is related to the changes of astrocyte activation in the MT and ACC, but there is no more powerful evidence to prove that the effect of electroacupuncture on IBS visceral hypersensitivity is mediated by astrocytes, or electroacupuncture plays a therapeutic effect through MT-ACC pathway. In the future, we will apply gene knockout animal models, fluorescence retrograde labeling, electrophysiology, optogenetics, and other techniques to provide more direct evidence for electroacupuncture to regulate the MT-ACC pathway of IBS visceral hypersensitivity. At the same time, the placebo effect of electroacupuncture should be excluded to clarify the uniqueness of electroacupuncture in the treatment with visceral pain. Although there are so many limitations, our research data have been able to support the conclusion.

## 5. Conclusions

In summary, this study found that GFAP gene and protein levels in MT and ACC of rats with IBS visceral hypersensitivity were upregulated and electroacupuncture could improve visceral hypersensitivity and downregulate GFAP levels in MT and ACC, revealing that reactive astrocytes in MT and ACC are involved in visceral hypersensitivity of IBS and thus electroacupuncture could alleviate IBS visceral hypersensitivity by inhibiting the activation of astrocytes in MT and ACC.

## Figures and Tables

**Table 1:** Abdominal withdrawal reflex (AWR) scoring criteria.

	
Score 0	No behavioral response to colorectal distension
Score 1	Immobile during colorectal distension and occasionally clicked the head at the onset of the stimulus
Score 2	A mild contraction of abdominal muscles, but no lifting of the abdomen off the platform
Score 3	A strong contraction of abdominal muscles and lifting of the abdomen off the platform, and no lifting of the pelvic structure off the platform
Score 4	Arching body and lifting of the pelvic structure and scrotum

## Data Availability

The initial data used to support the findings of this study are included within the article.
